# Invasive Intracranial Electroencephalogram (EEG) Monitoring for Epilepsy in the Pediatric Patient With a Shunt

**DOI:** 10.7759/cureus.35279

**Published:** 2023-02-21

**Authors:** Muhammad El Shatanofy, Katherine Hofmann, John S Myseros, William D Gaillard, Robert F Keating, Chima Oluigbo

**Affiliations:** 1 Neurosurgery, Children's National Hospital, Washington, USA; 2 Neurology, Children's National Hospital, Washington, USA

**Keywords:** pediatric hydrocephalus, subdural monitoring, non-infectious shunted hydrocephalus, stereotactic electroencephalography, invasive intracranial monitoring

## Abstract

The use of invasive intracranial electroencephalogram (EEG) monitoring in the patient with a cerebrospinal fluid (CSF) diversionary shunt presents a conundrum -- the presence of a percutaneous electrode passing into the intracranial compartment presents a pathway for entry of pathogens to which a chronically implanted device like a shunt is especially susceptible to infection.^ ^In this case report, we describe the clinical and radiological features, medical and surgical management, and treatment outcomes of pediatric patients with shunted hydrocephalus who underwent invasive intracranial monitoring over an eight-year period. Three cases of children undergoing invasive intracranial monitoring were included in this study. Invasive monitoring for each patient occurred over three to six days. In each case, invasive intracranial monitoring was completed successfully, without resulting infection or shunt malfunction. While the second procedure was complicated by the formation of a pneumocephalus, there was no associated midline shift, and invasive intracranial monitoring was completed without incidence. Each patient received further surgery that successfully reduced seizure frequency. This study suggests that, while children with CSF diversionary shunts are at an inherently increased risk for infection and other complications, invasive intracranial monitoring is a relatively safe and feasible option in these patients. Future studies should explore the optimal duration for intracranial monitoring in pediatric patients with chronically implanted devices.

## Introduction

Epilepsy is a chronic neurologic disorder categorized by unprovoked or reflex seizures [1-3]. While most children achieve good seizure control with anti-epileptic medications, some develop drug-resistant epilepsy defined as the failure of two tolerated and appropriate anti-seizure medications [4]. These children may benefit from surgical resection of the cortical seizure onset zone.

Surgical options for epilepsy range from focal resection of the epileptogenic cortex, such as the antero-mesial temporal lobe, to removing a portion of diseased cortex, through functional hemispherectomy or anterior corpus callosotomy [1]. Cortical resection requires precise identification of the cortical seizure onset zone. This may be achieved by non-invasive Phase 1 investigations such as brain MRI, fMRI, video EEG (vEEG), MEG, and FDG-PET scans. However, when these non-invasive investigations are insufficient to identify the seizure onset zone, intracranial EEG (iEEG) monitoring using subdural grids and strips, or stereotactic EEG (SEEG) depth electrodes may be indicated [4-9].

In a retrospective chart review of 189 patients with drug-resistant epilepsy, Van Gompel et al. found that subdural grid monitoring successfully identified the epileptogenic zone in 79% of sessions [9]. The major complication rate was 6.6%, including five infections and six hematomas [9]. This study found no correlation between risk for infection or complications with the duration of monitoring [9]. Similarly, Johnston et al. found no correlation between the risk of complications and the mean duration of monitoring or mean number of electrodes [10]. Regarding SEEG, a 2016 meta-analysis reported a SEEG insertion and monitoring complication rate of 1.3% [11].

The use of invasive iEEG monitoring in the patient with a cerebrospinal fluid (CSF) diversionary shunt presents a conundrum -- the presence of a percutaneous electrode passing into the intracranial compartment allows for entry of pathogens to implanted devices, which are especially susceptible to infection. Shunt infections are a common cause of shunt failure and associated neurological morbidity [3, 12-13]. Children with CSF shunts constitute a significant proportion of pediatric neurosurgeons’ clinical workload, with the global incidence of pediatric hydrocephalus requiring shunts estimated as over 90% [14]. There is also a higher incidence of epilepsy in patients with shunted hydrocephalus, with the incidence ranging from 20% to 50% [3]. This is likely due to underlying conditions, such as intracranial infections, intracranial hemorrhage, and severe head trauma that lead to these two pathologies. It is, therefore, not unexpected that some patients with shunted hydrocephalus also need surgical treatment for coexisting intractable epilepsy, including invasive iEEG monitoring.

To the best of our knowledge, no studies have explored the safety and utility of invasive intracranial monitoring for patients with shunted hydrocephalus. In this case report, we present the considerations in invasive intracranial monitoring in the child with shunted hydrocephalus and depict the principles, technical challenges, and considerations in these unique clinical scenarios.

## Case presentation

We retrospectively identified patients with shunted hydrocephalus who underwent invasive intracranial monitoring for drug-resistant epilepsy in the Department of Neurosurgery at Children’s National Hospital over an eight-year period. The following inclusion criteria were used: ages 1 month-17 years old, history of shunted hydrocephalus, and drug-resistant epilepsy defined as the failure of two tolerated and appropriate anti-seizure medications. Three cases were identified and presented in this study. The first two cases occurred in the same patient. Each operation for implantation of the iEEG monitoring device was performed by the same pediatric neurosurgeon.

Pre-operative studies included: patients’ seizure characteristics, pre-surgical neurological and neuropsychological assessment scores, EEG and ictal vEEG recordings, 3T epilepsy protocol, and when necessary, FDG-PET. Intra-operative data including electrophysiological monitoring data, electrocorticographic data, intra-operative cortical mapping data, number of SEEG electrodes, and location of subdural grids were also reviewed. Post-operative data included length of stay, surgical complications, location of subdural grids, results of extra-operative cortical mapping, and post-operative brain MRI or CT.

Over an eight-year period, two patients with shunted hydrocephalus and epilepsy underwent invasive monitoring procedures. Patient 1 underwent SEEG implantation and intracranial grid implantation. Patient 2 only underwent SEEG implantation. Both patients later received epilepsy surgery, where Patient 1 received palliative surgery (NeuroPace implantation) and Patient 2 received resection surgery. Both surgeries were successful and resulted in reduced seizure frequency for each patient.

Case 1 -- patient 1 (invasive intracranial monitoring procedure 1 - SEEG implantation)

A 12-year-old male with a history of congenital hydrocephalus, multiple ventriculoperitoneal (VP) shunt revisions, a large intracranial cyst, and partial agenesis of the corpus callosum, presented for pre-surgical evaluation of medically refractory epilepsy. Preliminary investigations included vEEG, FDG-PET scan, brain 3T MRI, genetic chromosome microarray, neuropsychological assessments, fMRI, and MEG. Prior vEEGs captured five electroclinical seizures of right hemisphere onset, predominantly in the right temporal area when compared to the frontoparietal regions. In three of these events, the patient displayed arrest of activity, staring, automatisms, and loss of contact with his surroundings. Brain MRI also demonstrated shunted mildly enlarged lateral ventricles consistent with his known history of corpus callosum hypogenesis and dysgenesis, as well as bilateral small hippocampi with abnormal architecture.

**Figure 1:**
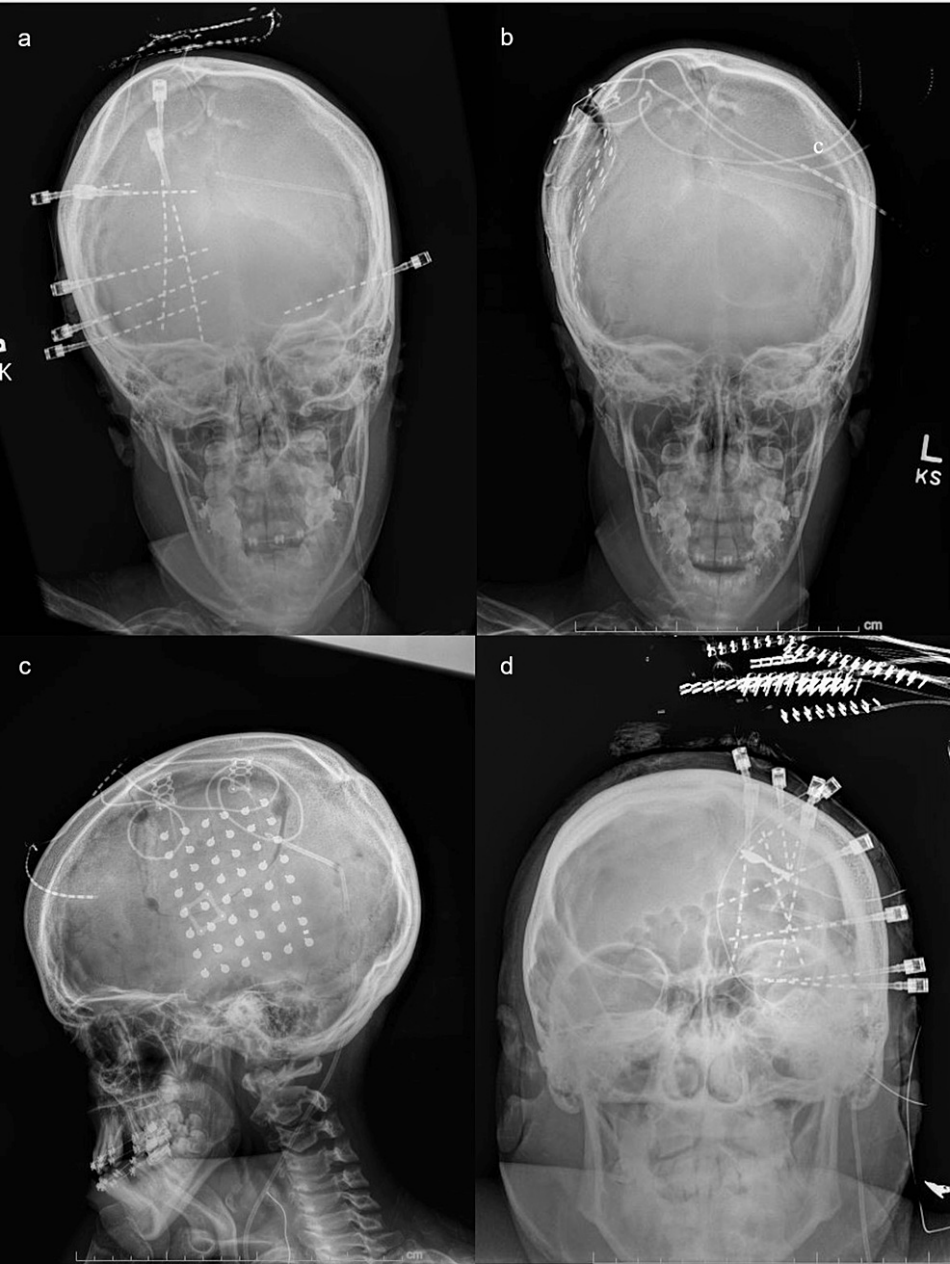
X-rays showing the location of invasive intracranial monitoring devices in three cases of shunted hydrocephalus. a. Patient 1 (invasive intracranial monitoring procedure 1 – SEEG implantation), eight depth electrodes placed in bilateral hemispheres with shunt in the left ventricle. b. Patient 1 (invasive intracranial monitoring procedure 2 – intracranial grid implantation), anteroposterior X-ray showing 48 contact subdural grids overlying the right frontoparietal area with left ventricular shunt. c. Patient 1 (invasive intracranial monitoring procedure 2 – intracranial grid implantation), lateral X-ray showing 28 contact subdural grids overlying the right frontoparietal area with left ventricular shunt d. Patient 2 (SEEG implantation), 10 depth electrodes positioned in the left hemisphere with left ventricular shunt in place. SEEG, stereotactic electroencephalogram

To further characterize the epileptogenic focus, this patient underwent invasive intracranial monitoring via bi-hemispheric SEEG depth electrode implantation using the ROSA stereotactic neurosurgical robot (Zimmer Biomet, Warsaw, IN). Eight total depth electrodes were placed in the following regions: right superior frontal gyrus-anterior insular, right superior frontal gyrus-medial orbital frontal, right middle frontal gyrus-anterior cingulate, right middle frontal gyrus-posterior prefrontal, right inferior frontal gyrus-prefrontal, right frontal MEG dipole, right hippocampus, and left hippocampus (Figure 1a).

The patient had rapid wean of seizure medications and, over a six-day period of invasive intracranial monitoring, had five electroclinical seizures, all of which were from the right frontal MEG dipole near the right motor cortex. The SEEG depth electrodes were safely removed and the results were discussed by a multidisciplinary epilepsy team. The team decided that because the seizures arose from an SEEG depth electrode close to the motor cortex, the patient would have to return for extra-operative grid-based brain mapping to determine the boundaries of the motor cortex and the proximity of this putative epileptogenic zone to the motor cortex. The patient had an uneventful recovery and was safely discharged home.

Case 2 -- patient 1 (invasive intracranial monitoring procedure 2 - intracranial grid implantation)

The same 12-year-old male presented post-SEEG for secondary invasive intracranial monitoring with a subdural grid to help define the epileptogenic focus of his drug-resistant seizures and its putative relationship to his motor cortex. The goal of additional monitoring was to determine whether to resect the epileptogenic focus, provided it did not involve eloquent sensorimotor cortex, or to implant a NeuroPace responsive neuro-stimulator (NeuroPace, MountainView, CA) if the motor cortex was involved. He underwent a SEEG-guided right craniotomy, and a 48-contact subdural grid was implanted centered where the right frontal MEG dipole SEEG depth electrode had been previously placed (Figure 1b,c).

**Figure 2:**
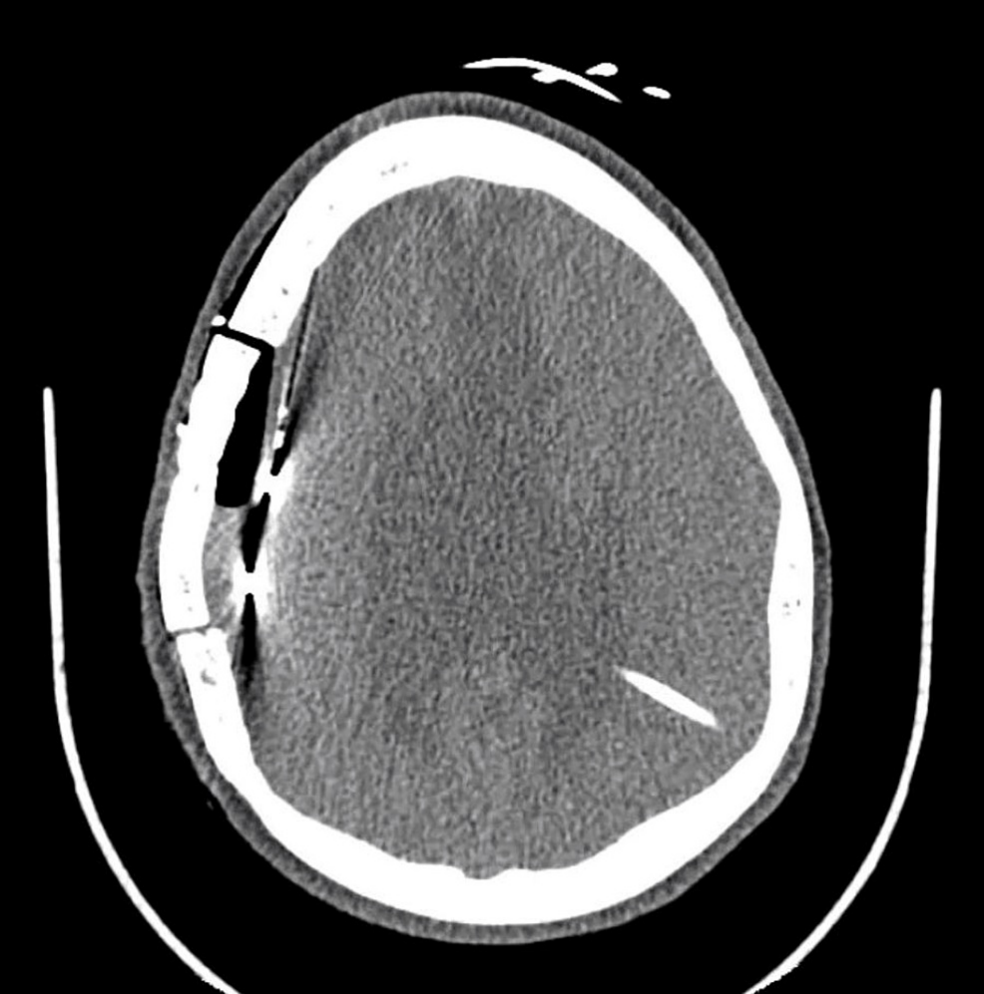
Axial non-contrast CT scan demonstrating a new extra-axial collection, approximately 12 mm in thickness, after placement of 48 contact subdural grids overlying the right frontoparietal area with left ventricular shunt in patient 1 (invasive intracranial monitoring procedure 2 – intracranial grid implantation).

Post-operatively, an axial CT of the brain demonstrated a new extra-axial collection approximately 12 mm in thickness. This collection was noted to contain mostly air and a small amount of dependent fluid (Figure 2). Notably, there was no significant associated midline shift or acute hemorrhage. The patient tolerated the procedure well and did not manifest any symptoms clinically correlating with these post-operative findings.

Over the three-day period of intracranial monitoring, the patient experienced three stereotypical electroclinical seizures originating from grid contacts corresponding to the face and hand motor cortex. Extra-operative grid-based stimulation of these contacts led to facial twitching, face and hand movement, as well as stereotypical seizures involving his face and hand. Collectively, these findings indicated that his seizures arose from the motor cortex and therefore would not be amenable to resection due to the expected neurological deficits. The decision was made to remove the grids and advise the patient to return for NeuroPace responsive neurostimulator implantation.

Subdural grid removal was uncomplicated and a head CT without contrast confirmed that the shunt catheter was stable in position and terminating near midline. At three weeks post-grid removal, the patient and his parents denied any fevers or symptoms suggesting shunt malfunction. Apart from his typical seizures, his postoperative recovery was uneventful.

The patient returned a few weeks later for the implantation of two NeuroPace responsive neuro-stimulators. He had a 50% improvement of his seizure frequency and enhanced overall quality of life.

Case 3 -- patient 2 (SEEG implantation)

A 17-year-old female with a medical history of refractory focal epilepsy in the setting of malformation of cortical development, cortical visual impairment, developmental delay, right spastic hemiparesis, and shunted hydrocephalus presented for SEEG. This patient was diagnosed with hydrocephalus in utero and had a shunt placed at 10 days old, which was revised at the age of 15 months and then several times over the next two years with subsequent change of the shunt valve to an adjustable pressure valve.

Initial investigation with vEEG captured five electroclinical seizures originating in the left frontal lobe and left temporal region. Typical seizure semiology involved eye fluttering, left head deviation, as well as motor activity of the right arm and leg. Brain MRI revealed multiple bilateral cerebral malformations of cortical development and corpus callosum dysgenesis with underdevelopment of the cerebral hemispheres, left greater than right, as well as a markedly small left hippocampus with abnormal architecture likely representing left mesial temporal sclerosis and or dysplasia. Functional MRI could not be done prior to EEG because the patient had a programmable shunt valve. Ultimately, the decision was made to place SEEG depth electrodes in the left hemisphere, including the left frontal, left temporal, left supplementary motor area (SMA), and left insular, as her semiology and vEEG data suggested left frontal or left temporal lobe seizure origin (Figure 1d).

One of the main concerns with pursuing stereotactic implantation of left hemispheric SEEG was the risk of shunt infection or shunt failure due to the prolonged presence of the subcutaneous depth electrodes. The parents were informed of the risks and agreed to proceed with the operation. Intraoperatively, 10 depth electrodes were stereotactically implanted within the left hemisphere with 78 total contacts. At day one post-operation, a head CT without contrast confirmed that the left parietal approach ventricular catheter was stable in position and that there was no significant change in ventricular size.

Over a four-day period of invasive intracranial monitoring, the patient had multiple clinical and electrographic seizures, indicating that her seizures originated from the left amygdala and left hippocampal depth electrodes. Five days after SEEG placement, sufficient evidence had been gathered and the electrodes were removed. Post-operative imaging was unremarkable.

At follow up one month later, the patient’s mother denied any fevers, incisional issues, or any symptoms suggestive of shunt malfunction. After five weeks, the patient underwent a left anterior lobectomy and amygdalohippocampectomy. Her surgery was uncomplicated, and she was discharged home on postoperative day 3. Four-and-a-half-weeks post-operatively, the patient denied any seizures, and did not demonstrate any signs or symptoms suggestive of increased intracranial pressure or shunt failure. However, five months post-operatively, the patient re-presented with one week of fatigue and increased ventricular size compared to five days prior on outpatient MRI. Shunt interrogation in the emergency department was concerning for shunt failure, so the patient was taken to the operating room for proximal shunt revision. The patient tolerated the procedure well and was discharged home the next day. She did not display further signs of shunt failure over the following 22 months.

## Discussion

Children with shunted hydrocephalus are at a higher risk of developing seizures than the general pediatric population [3, 12, 15]. In a longitudinal cohort study of 379 children with hydrocephalus, 23% developed epilepsy at a mean age of 2.7 years [16]. Epilepsy in children with hydrocephalus may occur because of brain injury due to hydrocephalus or as an indirect consequence from a hydrocephalus-related syndrome or surgical treatment, like CSF shunting [16]. When patients with epilepsy develop drug-resistance, defined as the failure of two tolerated and appropriate anti-seizure medications, iEEG may be pursued to determine candidacy for resection of the epileptogenic zone.

While the risks associated with invasive intracranial monitoring have previously been described in children with drug-refractory epilepsy, few studies have explored the outcomes of patients with shunted hydrocephalus [5, 9-10, 17-18]. This is important because, theoretically, children with shunted hydrocephalus are at a higher risk of shunt failure and infection during intracranial procedures because of the presence of a percutaneous intracranial indwelling device. In this study, we describe three cases where intracranial monitoring was used to identify the epileptogenic focus in children with VP shunts. Each case was uncomplicated, and the patients recovered without signs of infection or shunt malfunction.

In the first case, depth electrodes were implanted for six days, comparable to the mean duration of invasive intracranial monitoring in all pediatric patients [18]. While the long-term effects of SEEG implantation in pediatric patients remain unclear, several studies suggest that pediatric patients can tolerate up to four weeks of recording [5]. A retrospective study by Johnston et al. found that the duration of monitoring was not significantly correlated with the risk of complications, even when monitoring continued for 21 days [10]. This presents the question of whether children with shunted hydrocephalus can also tolerate intracranial monitoring for up to three weeks, which requires examination of the potential benefits and risks of monitoring in this unique population. In a retrospective review of 317 electrode implantation procedures, the overall complication rate for intracranial monitoring was consistently around 9%, with less than 1% of patients showing permanent deficits [19]. Common adverse events associated with intracranial electrode placement included extra-axial collection (19.6%), hemorrhage (16.4%), infection (5.7%), contusion (4.4%), edema (2.5%), infarct (2.2%), and CSF leak (0.9%) [19]. With regard to non-hemorrhagic extra-axial collections, 1.9% of cases were clinically significant with two cases resulting in transient hemiparesis, two cases resulting in aphasia, and one case leading to transient but decreased arousability [19]. In children with hydrocephalus, these risks are potentially augmented by the presence of a foreign body that is the VP shunt.

Another risk associated with invasive intracranial monitoring in patients with shunted hydrocephalus is the risk of hemispheric ventricular collapse. Hemispheric ventricular collapse may occur due to exposure of the brain to atmospheric pressure through the percutaneous intracranial indwelling device. In catastrophic scenarios, this can lead to midline shift, brain herniation, and sudden death. In milder cases, the pressure gradient and subsequent ventricular collapse can promote the formation of a subdural collection or hematoma between the subdural grids and pial surface of the brain. In the absence of direct contact with the brain, signal sensitivity can decrease, resulting in decreased quality of intracranial monitoring [8].

In this study, we observed one case where invasive intracranial monitoring resulted in the formation of a small extra-axial collection of air and fluid (Figure 2). Intracranial monitoring continued for three days, and the patient remained asymptomatic without signs of infection or shunt malfunction. Post-operative head CT showed no acute hemorrhage or midline shift. Additionally, the quality of the signal from the electrodes remained consistent monitoring. This case suggests that, while invasive intracranial monitoring may increase the pressure gradient in the setting of VP shunting, it remains a feasible option for identifying the epileptogenic zone in children with hydrocephalus. Future work should explore whether the pressure differential increases with longer periods of monitoring.

Future work should also explore the risk of shunt obstruction following invasive intracranial monitoring. To the best of our knowledge, no studies have previously quantified the risk of shunt malfunction in the setting of SEEGs. In a retrospective study on the risk of shunt malfunction in pediatric patients with existing shunts who underwent elective intradural operations, 12.8% of patients experienced shunt malfunctions within 90 days of elective intradural surgery with a median time to failure of nine days [20]. Risk factors associated with shunt malfunction included an intraventricular surgical approach, shorter time since shunt related surgery, and young age. In our study, we noted one patient who experienced a shunt malfunction eight months after invasive intracranial monitoring with depth electrodes, which was also five months after her left anterior lobectomy and amygdalohippocampectomy. Given the prolonged time to shunt failure, and given its temporal relationship to her last surgery of temporal lobectomy, it appears that the failure was most likely related to the temporal lobectomy, which is a surgery associated with entry into the ventricular system and extensive brain dissection and resection with resultant release of significant amounts of inflammatory elements into the cerebrospinal fluid, which are all factors that increase the risk for shunt failure.

Limitations to this study exist. First, we were limited by the small sample size. Due to the strict inclusion criteria of this study, only three cases of patients with shunted hydrocephalus who underwent invasive intracranial monitoring for mapping of the epileptogenic zone were identified; however, to the best of our knowledge, this is the largest cohort of its kind. Because of the small sample size, we had limited statistical power to perform any analyses. Second, this study is limited by its use of existing data. Surgical procedures and EEG results were reliably documented, but subjective information in progress notes may not have addressed immediate concerns or complications throughout the monitoring period. This may have resulted in the underestimation of mild and subclinical neurological complications. Finally, as with any observational study, our ability to determine causality between the duration of monitoring and infection or other complication risks was limited.

## Conclusions

Children with shunted hydrocephalus and drug-resistant epilepsy may benefit from invasive intracranial monitoring to help identify the epileptogenic zone and plan for surgical intervention. To the best of our knowledge, no studies have previously described the risks of infection or other complications in children with shunted hydrocephalus undergoing invasive intracranial monitoring. This study uses three cases to depict the principles, technical challenges, and considerations in these unique clinical scenarios. Invasive intracranial monitoring was safely performed for a duration of up to six days, comparable to the duration of invasive intracranial monitoring for patients without VP shunts. Future studies should explore the optimal duration for monitoring in this population at an inherently increased risk of infection, and larger sample sizes should be investigated.
